# The Effects of a Biomimetic Hybrid Meso- and Nano-Scale Surface Topography on Blood and Protein Recruitment in a Computational Fluid Dynamics Implant Model

**DOI:** 10.3390/biomimetics8040376

**Published:** 2023-08-18

**Authors:** Hiroaki Kitajima, Makoto Hirota, Kohei Osawa, Toshinori Iwai, Kenji Mitsudo, Juri Saruta, Takahiro Ogawa

**Affiliations:** 1 Weintraub Center for Reconstructive Biotechnology, UCLA School of Dentistry, Los Angeles, CA 90095-1668, USAmhirota@yokohama-cu.ac.jp (M.H.); saruta@kdu.ac.jp (J.S.); 2 Division of Regenerative and Reconstructive Sciences, UCLA School of Dentistry, Los Angeles, CA 90095-1668, USA; 3 Department of Oral and Maxillofacial Surgery, Graduate School of Medicine, Yokohama City University, 3-9 Fukuura, Kanazawa-ku, Yokohama 236-0004, Japan; t166016e@yokohama-cu.ac.jp (K.O.); iwai104@yokohama-cu.ac.jp (T.I.); mitsudo@yokohama-cu.ac.jp (K.M.); 4 Department of Oral and Maxillofacial Surgery/Orthodontics, Yokohama City University Medical Center, 4-57 Urafune-cho, Minami-ku, Yokohama 232-0024, Japan; 5 Department of Education Planning, School of Dentistry, Kanagawa Dental University, 82 Inaoka, Yokosuka 238-8580, Japan

**Keywords:** bone-implant integration, computational fluid dynamics (CFD), osseointegration, titanium implant, zirconia implant

## Abstract

The mechanisms underlying bone-implant integration, or osseointegration, are still incompletely understood, in particular how blood and proteins are recruited to implant surfaces. The objective of this study was to visualize and quantify the flow of blood and the model protein fibrinogen using a computational fluid dynamics (CFD) implant model. Implants with screws were designed with three different surface topographies: (1) amorphous, (2) nano-trabecular, and (3) hybrid meso-spikes and nano-trabeculae. The implant with nano-topography recruited more blood and fibrinogen to the implant interface than the amorphous implant. Implants with hybrid topography further increased recruitment, with particularly efficient recruitment from the thread area to the interface. Blood movement significantly slowed at the implant interface compared with the thread area for all implants. The blood velocity at the interface was 3- and 4-fold lower for the hybrid topography compared with the nano-topography and amorphous surfaces, respectively. Thus, this study for the first time provides insights into how different implant surfaces regulate blood dynamics and the potential advantages of surface texturization in blood and protein recruitment and retention. In particular, co-texturization with a hybrid meso- and nano-topography created the most favorable microenvironment. The established CFD model is simple, low-cost, and expected to be useful for a wide range of studies designing and optimizing implants at the macro and micro levels.

## 1. Introduction

Developing a more osteoconductive endosseous implant surface than that of “microrough” implant surfaces has proven difficult due to the well-established capability of microrough titanium surfaces [1,2,3,4,5,6,7,8], technical challenges of nanotechnology [3,9,10,11,12,13,14,15,16,17,18,19,20,21], potential biological limits of bone generation [9,10,11,13,14,22], restricted use of different types of materials other than titanium [23,24,25], and cost–performance ratio consideration [3,10,11,15,23,25,26]. Microrough surfaces on titanium, titanium alloy, or zirconia with acid-etched [7,27,28,29], sandblasted [2], oxidized [30,31], alkaline-treated [23,25], or plasma-sprayed surfaces [30,31,32] are used in most dental and orthopedic implants. A typical microrough topography is a formation of compartments made of ~5 mm-interval peaks and valleys and, compared with relatively smooth surfaces like machined surfaces, not only increase mechanical interlocking between the implant and bone but also promote the differentiation of osteogenic cells growing on the surface and increase the mechanical quality of de novo bone [4,6,7,27,28,32,33,34,35,36,37]. Together, these properties accelerate and improve bone-implant integration, or osseointegration. Among the reasons why no titanium or zirconia surface that outperforms microrough surfaces has been developed, the most significant challenge is the technical difficulty in creating the defined roughness at the meso- (between 10 and 500 mm) and nano-scales, or indeed adding roughness at a new scale to the existing micro-scale. Some advances in hybrid micro- and nano-surfaces have yet to replace microrough surfaces or remain experimental due to their high cost and technical complexity [3,11,13,14,15,17,18,20,23,26,29].

Recent advances in laser technology have allowed the simultaneous generation of meso- and nano-topographies on metallic surfaces [38,39,40,41,42,43,44]. Particular studies utilizing solid-state laser etching created a unique meso-and-nano hybrid zirconia surface on which the meso-topography mimics “cactus-like” oscillating spikes, while the nano-topography consists of dense, nodular protrusions, 100–300 nm in diameter, resembling trabecular bone [45,46]. The cactus topography drastically increased the surface area of the implant as well as physical mechanical interlocking between the implant and bone [46]. Indeed, implant primary stability or the implant stability immediately after implant placement is considered the most pertinent factor for successful osseointegration [47,48], to which the enhanced meso-scale topography of the cactus zirconia may have contributed. The increased surface area also contributed to an increase in the number of attaching cells [46]. The nano-topography promoted the differentiation of osteogenic cells recruited to and growing on the surface [45,46]. In vivo studies demonstrated that the biomechanical strength of osseointegration is considerably increased for the meso–nano hybrid zirconia compared with machined zirconia and even higher than micro-roughened titanium implants [46,49,50]. However, these documented mechanisms may not fully explain the advantages of the meso- and nano-hybrid surfaces. More importantly, in the general field of implant science and bone-and-implant integration, understanding biologic events other than the growth and function of osteogenic cells on implant surfaces is critically lacking. In particular, it is unknown how cells and proteins necessary for osseointegration are recruited to implant surfaces.

The recent application of computational fluid dynamics (CFD) to implant science has provided a novel approach to understanding the mechanisms of osseointegration and the effect of implant surface modification. For instance, hydrophilic implant surfaces significantly alter blood flow around the implant and increase the recruitment of blood and protein to areas within macroscopic threads, particularly to the implant interface, compared with hydrophobic implant surfaces [51,52]. These in silico approaches not only provided new knowledge about how osseointegration is established but also may allow the rational design and optimization of implants at the macro- and micro-scales for improved blood and protein recruitment. By exploiting a CFD model, this study attempts to examine the effects of meso- and nano-scale topography on the blood and protein dynamics. In particular, we utilized the recently created hybrid zirconia with meso-spikes and nano-trabeculae as a CFD model. Therefore, the objective of this study was to examine the density, speed, direction, and other dynamic behaviors of blood and proteins around screw-shaped implants with (1) amorphous surfaces, (2) nano-trabecular topography, and (3) hybrid meso-spike and nano-trabecular topography. We selected fibrinogen as a model protein due to its crucial role in bone healing. The null hypothesis tested was that the difference in surface topography does not influence the blood and protein flow around implants.

## 2. Materials and Methods

### 2.1. Creation of a Zirconia Surface with Meso–Nano Hybrid Topography

To design and establish the validity of the CFD models, we created a hybrid meso- and nano-scale topography using zirconia. First, experimental samples of zirconia in disk form (20 mm diameter, 1.5 mm thickness) were prepared from zirconia oxide powder with 3 mol% yttria oxide (3Y-TZP, Tosoh, Tokyo, Japan) by injection molding, followed by sintering at 1400 °C. The sintered yttria-stabilized tetragonal zirconia polycrystal (Y-TZP) specimens were then surface-textured by solid-stage laser treatment. The laser etching was originally developed to carve grooves with hemispherical bottom surfaces [38] and was used in this study in a crisscrossing direction to carve cactus-like meso-spikes, as reported previously [46]. The laser was adjusted to create 40 μm high, 50 μm wide spikes. This meso-topography was proven to show the highest osteoconductivity in previous studies by promoting cell attachment and maximizing osseointegration [38,45,46]. A previous study showed that the standard deviations of the dimensional measurements and various surface roughness parameters were less than 5%, highlighting the precision of the laser texturing [45]. Simultaneously, laser-etched zirconia was expected to show trabeculae-like nano-nodular structures by self-assembly all over the meso-scale cactus spiles [38]. The detail protocol of laser etching, such as the source, energy, wavelength, frequency of laser, is proprietary. X-ray photoelectron spectroscopy conducted in previous studies showed that the zirconia surface consisted of elements of zirconium, yttrium, oxygen, hafnium, and carbon [38].

To further verify the viability of the laser technology, a screw-shaped implant (11 mm length, 4 mm diameter) representing a standard sized and shaped dental implant was injection-molded with Y-TZP with macroscopic helical threads, and then laser-etched to create the hybrid meso- and nano-topography. The size of the meso-spikes was the same as the one on zirconia disks. All specimens were supplied from Nantoh Co., Ltd. (Numazu, Japan). The morphology of the specimens was assessed using scanning electron microscopy (SEM; Nova 230 Nano SEM, FEI, Hillsboro, OR, USA) and an optical profilometer with a 50× lens (VK-X110, Keyence, Itasca, IL, USA) for three-dimensional (3D) imaging in the area of 282.9 μm × 202.0 μm. The resolution of the profilometer was 0.005 μm and 0.01 μm for the vertical and horizontal detection, respectively, while the accuracy for repeated measurements was 0.02 μm and 0.03 μm along the vertical and horizontal direction, respectively. Quantitative analysis was conducted for surface roughness and the following parameters were calculated: the arithmetical mean height (Sa) defined as the extension of Ra (arithmetical mean height of a line) to a surface, expressing the difference in height of each point compared to the arithmetical mean of the surface; the maximum height (Sz) defined as the sum of the largest peak height value and the largest pit depth value within the defined area; the developed interfacial area ratio (Sdr) expressed as the percentage of the definition area’s additional surface area contributed by the texture as compared to the planar definition area.

### 2.2. Computational Fluid Dynamics (CFD) Implant Model

**Figure 1:**
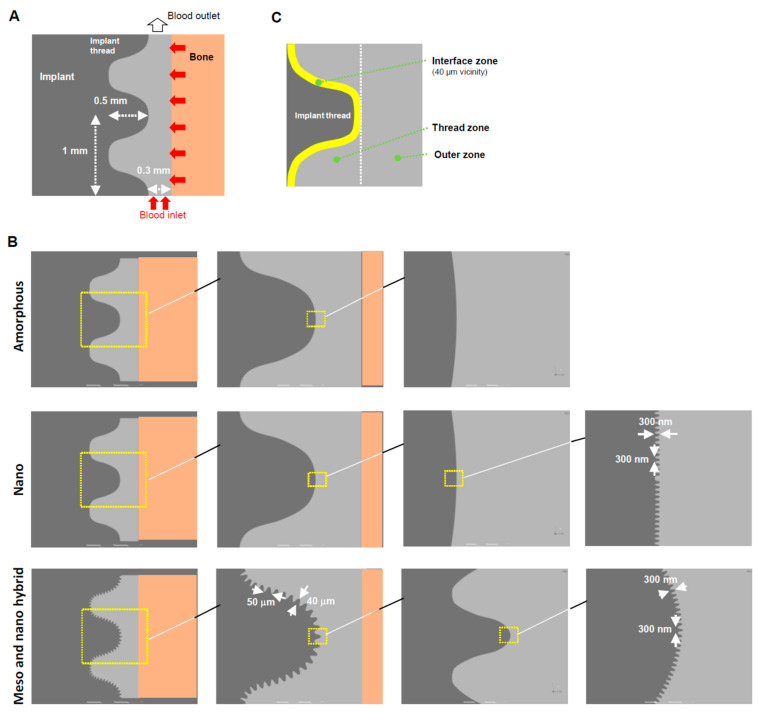
A computational fluid dynamics (CFD) model designed for a screw-shaped implant. (**A**) Dimensions and design of an implant, blood area, and boundary conditions. (**B**) Modeling of three different surface topographies. (**C**) Three different zones defined around an implant.

A geometric model for implants was created using ANSYS Design Modeler (2019 R1, ANSYS Inc., Canonsburg, PA, USA) to simulate the environment around the implants, as reported elsewhere [51,52] (Figure 1A). Three different topographies were modeled: (1) an amorphous surface with no projections or structure; (2) a nano-trabecular surface with nodular structures (300 nm height, 300 nm width); and (3) a hybrid meso- and nano-surface with a combination of meso-scale spikes (40 μm height, 50 μm width) and nano-nodules (300 nm height, 300 nm width) (Figure 1B). As mentioned earlier, this meso-scale topography was chosen based on the previous studies that optimized the morphology for the highest osteoconductivity [38,45,46]. The model, boundaries, and three different zones (interface, thread, and outer zones) were designed following the methods reported elsewhere (Figure 1C) [52]. The established analytical approach was used, including the volumes of the fraction model, species transport model, fluid properties, and numerical conditions [52].

## 3. Results

### 3.1. Demonstration of Hybrid Meso–Nano Surface Creation

**Figure 2:**
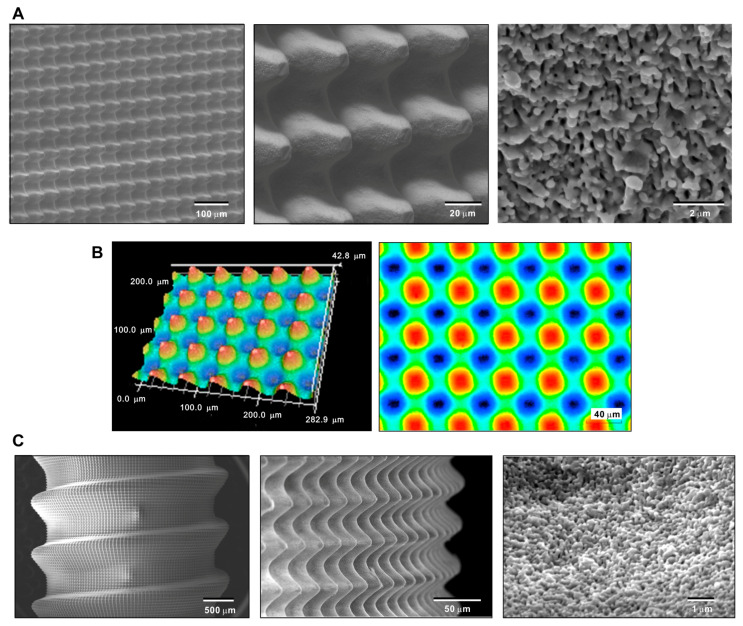
Creating the hybrid textured zirconia surface consisting of cactus-like meso-scale spikes and trabeculae-like nano-scale nodules. Low- and high-magnification SEM (**A**) and 3D optical (**B**) images of the laser-etched zirconia. (**C**) SEM images of a laser-etched dental implant prototype made of zirconia.

Because a CFD model was to be created based on the actual experimental specimens, it was crucial to demonstrate that specimens with a hybrid morphology can be made. Therefore, we first demonstrated the creation of a biomimetic meso–nano hybrid surface by laser etching a flat disk zirconia. As shown in the low-magnification images, accurate, organized arrays of meso-scale 40 μm oscillating peaks and valleys were clearly formed (Figure 2A). High-magnification images showed the even and uniform formation of nano-scale trabeculae-like nodular structures (100–300 nm width) all over the meso-peaks, inclines, and valleys (right panel in Figure 2B). In addition, 3D surface imaging confirmed the formation of the meso-peaks and quantitative analysis showed that the arithmetical mean height (Sa), maximum height (Sz), and developed interfacial area ratio (Sdr) were 7.71 μm, 42.8 μm, and 2.83 μm, respectively. The width of a meso-spike was approximately 50 μm, as shown in the 2D projection (right panel in Figure 2B).

After confirming the feasibility of creating hybrid topography on flat zirconia surfaces, we determined whether similar topography can be created on a 3D complex shape mimicking dental implants. SEM revealed a vivid, uniform, and seamless creation of arrays of meso-spikes all over the peaks, flanks, and valleys of implant threads, similar to the appearance observed on the flat zirconia disk. Nano-trabeculae were also confirmed on the dental implant-shaped zirconia. These qualitative and quantitative morphological assessments of the laser-textured zirconia validated the design and dimensions of the CFD model to be created.

### 3.2. Blood Plasma Flow Visualization and Quantification around Implants with Three Different Surface Topographies

**Figure 3:**
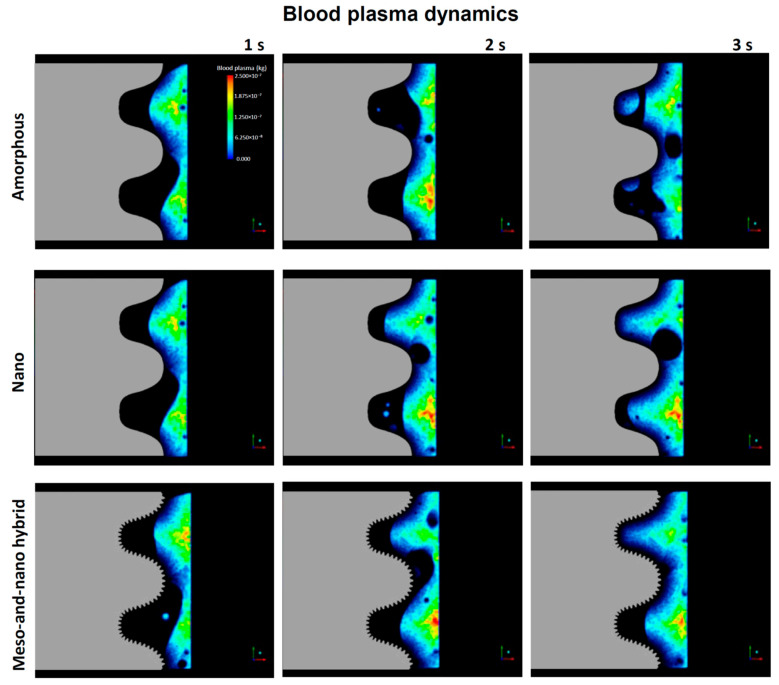
Blood dynamics visualized by color mapping the blood plasma density. Implants with three different surface topographies are compared.

As mentioned in the Introduction, little information is available on how cells and proteins are recruited to implant surfaces. It was reasonable to begin with an overall qualitative assessment of blood flow in the implant model. To visualize blood flow, we conducted color mapping of blood plasma density around the implants with either an amorphous surface, nano-trabeculae, or a hybrid surface from the commencement of blood flow up to 3 s. In the model, blood entered at the apex of the implant and side-wall of the bone and then exited upwards, as shown in Figure 1A. As shown in the color maps (Figure 3), the plasma did not infiltrate into the implant threads of all the implants by 1 s, leaving voids in the implant threads and at the interface. Blood infiltration into the threads progressed from 1 to 3 s regardless of the surface topography. However, at 2 and 3 s, blood infiltration was more advanced in the threads of nano-trabecular and meso–nano hybrid surfaces compared with the amorphous surface.

**Figure 4:**
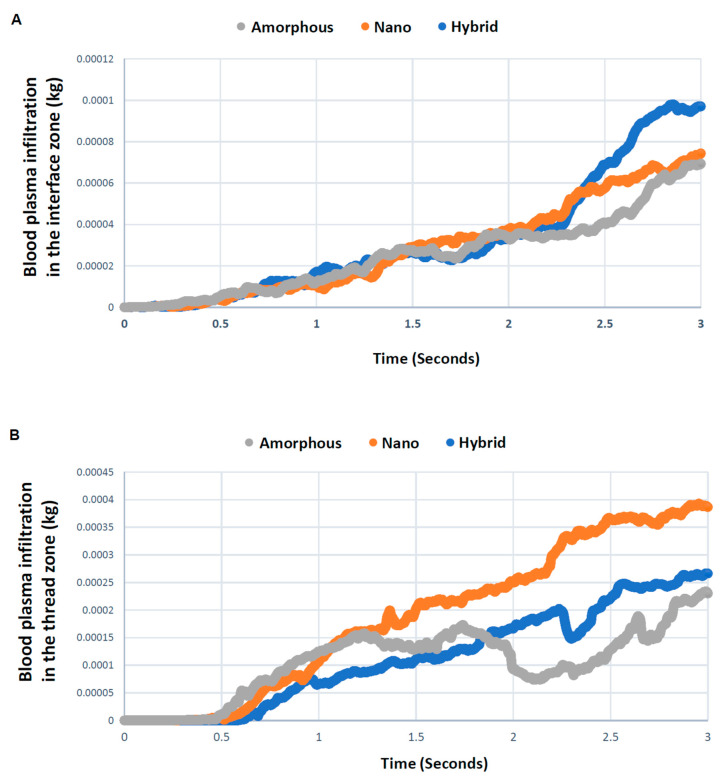
Quantitative assessment of time-dependent blood plasma quantity infiltrated into each of the interface (**A**) and thread (**B**) zones.

The blood plasma color mapping was useful but cross-sectional. Therefore, we next quantitatively assessed blood plasma in the interface and thread zones along the timeline. As shown in Figure 4A, blood plasma density in the interface zone increased for all three implant models over time. After 2 s, the plasma density varied significantly according to the surface topography, with the hybrid meso–nano surface increasing most rapidly, followed by the nano-trabecular surface. Approximately 40% more plasma was present at the hybrid interface than the amorphous and nano-trabecular surfaces at the end of the study period. In the thread zone, the plasma density was highest for the nano-trabecular surface at 3 s, followed by the hybrid surface (Figure 4B). These results indicated the surface topography-dependent and zone-specific differences in blood infiltration.

### 3.3. Direction and Velocity of Whole Blood Flow

**Figure 5:**
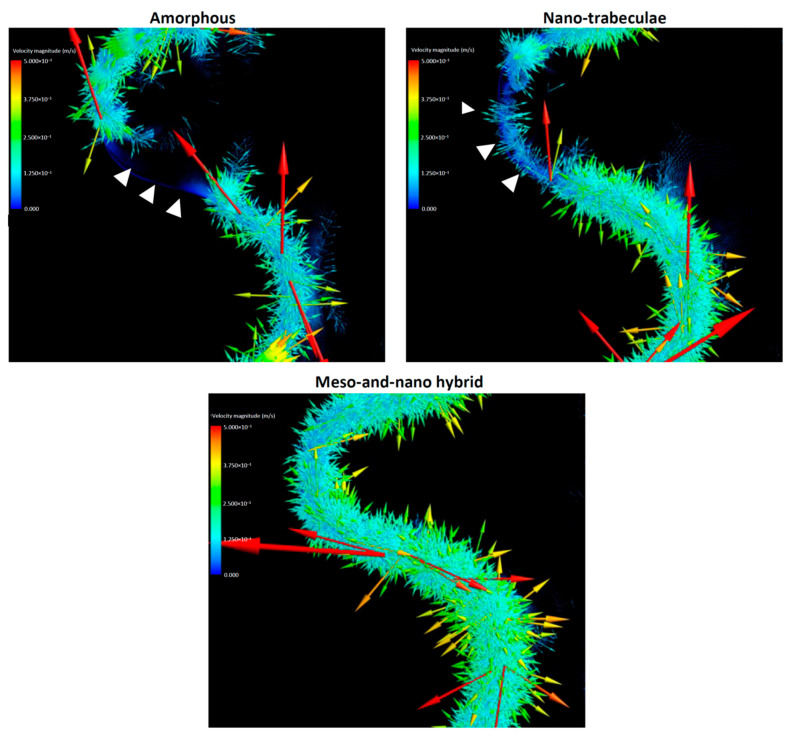
Vector color mapping for blood flow around three different surfaces. Each vector represents the direction and speed of the cell meshed in the domain. Refer to the main text for symbols.

The results of the blood mapping and density analyses simply showed the localization of blood plasma but not the dynamics. In particular, it was important to determine if the blood movement was static, slow, or fast as well as the direction of the movement, because how much cells and proteins are retained at the implant interface potentially depends on these dynamic parameters. Therefore, we evaluated the vector field formation across the different surfaces. Vector color mapping uncovered more robust vector formation at the implant interface than in the thread or outer zones for all implants and revealed considerable differences among the three different surfaces (Figure 5). Vectors were most dense at the hybrid interface and least dense at the amorphous interface. The particular area of the interface contained no or only sparse vectors around the amorphous and nano-trabecular surfaces (white arrowheads in top panels, Figure 5). As indicated by the majority of high-magnitude red vectors pointing vertically upwards (top panels, Figure 5), blood flow was, in general, upbound around amorphous and nano-trabecular interfaces, whereas all the red vectors were side-bound or slightly downbound around the hybrid meso–nano surface, indicating that the blood flow had slowed down or even stalled exclusively around the hybrid topography.

**Figure 6:**
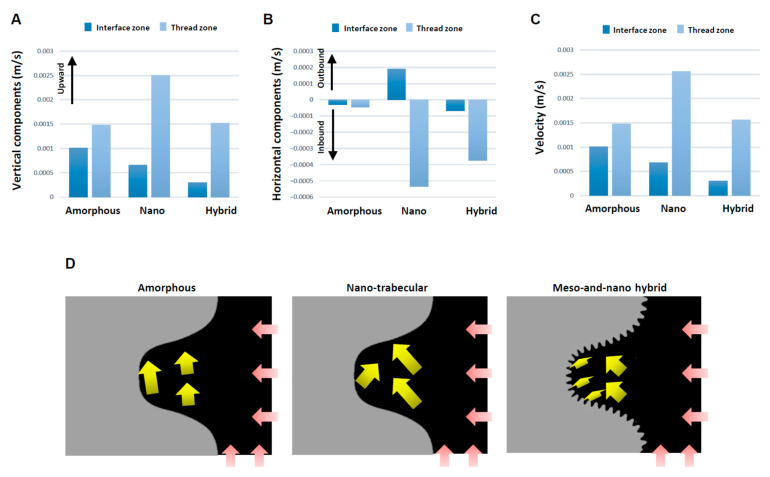
Evaluation for the direction and speed of whole blood flow. Vertical (**A**) and horizontal (**B**) components of averaged vectors of the blood field. Histograms are shown for the interface and thread zones and presented to compare the three different topographies. (**C**) Velocity of whole blood flow calculated from the averaged vectors. (**D**) Diagram of the whole blood flow (yellow arrows) described based on mean values of the vectors. Pink arrows indicate blood inlet.

These qualitative findings needed to be confirmed by quantitative assessment. Also, vectors need to be separated into vertical and horizontal components for the precise assessment of the direction and speed of blood movement. Quantitative assessment of the blood vectors showed that the averaged vertical component of the vectors in the interface zone was upbound for all the implants but lowest for the hybrid surface and highest for the amorphous surface (Figure 6A). The vertical component of the vectors in the thread zone was also upbound and higher than in the interface zone for all implants. The thread zone vertical vector was lower for the amorphous and hybrid surfaces than the nano-trabecular surface.

The horizontal components of the vector also differed significantly for the different surfaces (Figure 6B). Higher magnitude inbound vectors were found both in the interface and thread zones exclusively for the hybrid surface, while the vector was outbound at the nano-trabecular interface. Total velocity combined with the vertical and horizontal components was considerably small at the interface zone of the hybrid surface (Figure 6C), with a 3- and 4-fold difference compared with the nano-trabecular and amorphous surfaces, respectively. The velocity in the thread zone was slow for the amorphous and hybrid surfaces. The results of these averaged directions and magnitudes are schematically summarized in Figure 6D.

### 3.4. Fibrinogen Flow Visualization around Three Different Implants

**Figure 7:**
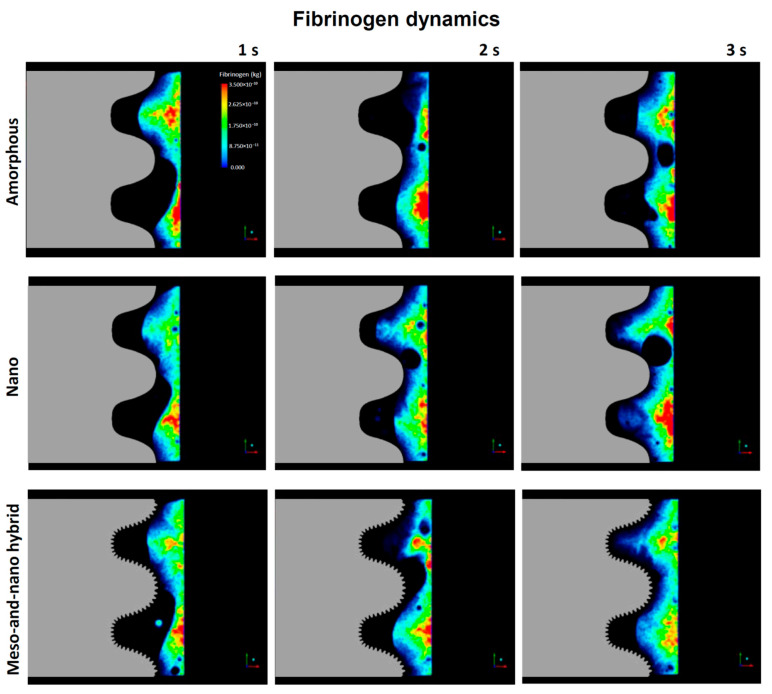
Fibrinogen dynamics regulated by three different topographies.

The blood movement does not necessarily represent the movement of cells and proteins that should be recruited to implant surfaces to initiate osseointegration. Therefore, we next examined the dynamics of fibrinogen as a model protein, starting with color mapping (Figure 7). Fibrinogen dynamics mimicked blood dynamics (Figure 3), in that the infiltration into the thread zone progressed over time. However, the speed of infiltration seemed to be slower than the blood, implying a time gap between the blood and proteins. Especially, there was a significant delay in thread zone filling for the amorphous surface compared with the nano-trabecular and hybrid surfaces.

**Figure 8:**
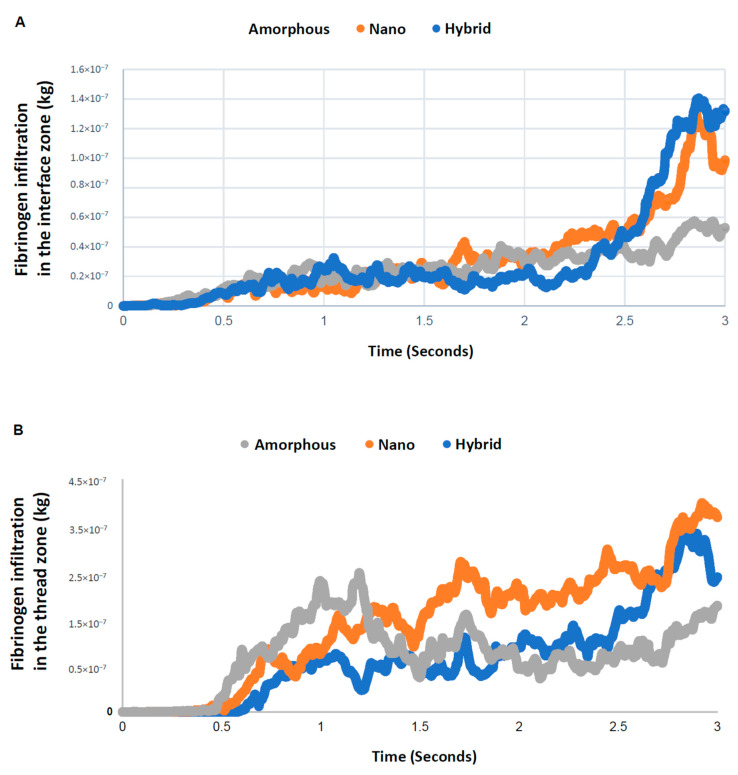
Quantitative assessment of time-dependent fibrinogen quantity infiltrated into each of the interface (**A**) and thread (**B**) zones.

A time-dependent plot of the quantity of fibrinogen in the interface zone showed an increase over time for all surfaces, with a particular surge after 2.5 s for the nano-trabecular and hybrid surfaces (Figure 8). Fibrinogen infiltration into the interface zone at 3 s was the highest for the hybrid surface and lowest for the amorphous surface, with a 2.5-fold difference. In the thread zone, the fibrinogen density was the highest for the nano-trabecular surface, followed by the hybrid surface.

### 3.5. Blood and Protein Recruitment Efficiency to the Implant Interface

**Figure 9:**
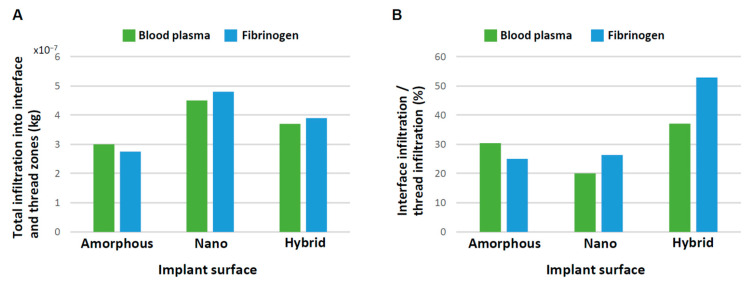
Efficiency of blood and protein recruitment to the implant interface. (**A**) Quantity of blood and fibrinogen infiltrating into both the interface and thread zones at 3 s. (**B**) Quantity of blood and fibrinogen infiltrating into the interface zone relative to into the thread zone.

Although we have analyzed the progressive change in blood and protein infiltration into the interface and thread zones, the total amount of their recruitment throughout the study time remained unknown. Because we believed the recruitment of blood and protein was (1) to the bay of the implant threads, and (2) immediately adjacent to the implant surface, which is particularly crucial for osseointegration, we analyzed the total quantity of blood plasma and protein infiltrating into both the thread and interface zones as well as the percentage of infiltration into the interface zone relative to that into the thread zone. The total infiltration at 3 s was highest for the nano-surface and lowest for the amorphous surface both for blood plasma and fibrinogen (Figure 9A). Very interestingly, the percentage of interfacial infiltration was the highest around the hybrid surface both for blood plasma and fibrinogen, demonstrating a particularly remarkable increase in fibrinogen (Figure 9B). The efficiency of fibrinogen recruitment to the hybrid interface was over twice that of the other two implant interfaces.

### 3.6. Overall Characterization of Blood and Protein Dynamics

The blood and protein dynamics around three different surfaces are summarized in Table 1.

## 4. Discussion

This is the first study exploring blood and protein flow around implants with different surface topographies. We revealed that surface topography drastically altered the distribution and density of blood and protein as well as their speed and direction of movement. In doing so, we showcase a new surface functionalization that alters the peri-implant microenvironment. Specifically, the presence of nano-trabeculae, regardless of the presence or absence of meso-spikes, promoted the recruitment of blood and fibrinogen into the implant thread compared with an amorphous surface by as much as 25–65% (Figure 9A). The presence of meso-spikes with nano-trabeculae slightly attenuated this enhanced recruitment. However, more importantly, the hybrid meso-nano surface recruited blood and fibrinogen to its interface more effectively than the amorphous surface and the surface with nano-trabeculae alone (Figure 9B), resulting in the highest density of blood plasma and fibrinogen at the hybrid interface.

We postulate that both the speed and density of blood and protein flow are crucial determinants of the subsequent biological outcome around implants. The slower the blood flow, the more blood components and proteins may persist at the implant interface, facilitating protein and cell attachment to the implant surface. Considering this hypothesis, this study revealed that the velocity of blood flow, as represented by the total vector magnitude, was significantly regulated by surface topography (Figure 6C,D). In the interface zone, the hybrid surface slowed the blood flow by 3- and 4-fold compared with the nano-trabecular and amorphous surfaces, respectively. Even the nano-trabecular topography alone significantly slowed the blood flow compared with the amorphous surface. Such a stagnating effect of blood was primarily observed in the vertical components of the vectors (Figure 6A,D). Individual vector visualization by color mapping indeed corroborated the result of the average components of the vectors. As depicted by the down- and side-bound red arrows rather than upbound ones for other surfaces (Figure 5), there were major down- and side-bound blood streams exclusively at the hybrid interface, indicating that the vertical, upbound blood stream was neutralized or minimal in the proximity of the hybrid surface. Furthermore, denser and more extensive vector formation at the hybrid interface compared with the other two surfaces consisted of vectors of conflicting direction, which may have neutralized the upward momentum of inflowing blood and slowed blood movement. Together, the unique meso-spikes created and tested in the present study may induce a bay effect in recruiting and retaining blood and proteins within the meso-habitat. Interestingly, the blood speed was faster in the nano-trabecular thread zone compared with the other two surfaces. Nano-topography alone may accelerate blood flow, which is attenuated or negated by the co-existence of meso-topography, i.e., the meso-effect overrides the nano-effect.

The results of the horizontal components of blood vectors were consistent with our hypothesis that all average vectors should be inbound or toward the implant surface, except those in the nano-trabecular interface (Figure 6B,D). The inbound vectors were the result of blood infiltration into the implant threads and continued to the interface zone, except for the nano-trabecular surface. This natural influx, although only currently evidenced by in silico simulations, can be considered a benefit of the screw-shaped structure and favorable for carrying blood, proteins, and cells to the area of greatest importance for osseointegration. Inbound movement at the interface and thread zones was enhanced substantially by the hybrid surface compared with the amorphous surface (Figure 6B), indicating increased blood recruitment and confirming the increased density of blood plasma and fibrinogen. The outbound average vector was exclusive to the nano-trabecular interface, and further studies are now required to explain the underlying mechanism.

The mechanism of osseointegration, particularly contact osteogenesis occurring around modern micro-rough implant surfaces, is not fully understood [5,27,53,54]. Regardless of the presence or absence of nano-topography or meso-spikes, this study provides novel, valuable information about the biological process of osseointegration. First, blood and protein flow was significantly slower in the interface zone than in the thread zone for all three surface groups tested. Second, blood vector formation at the implant interface was poly-directional, and thereby neutralized and negated for all surfaces. This biological phenomenon facilitates cells and proteins to settle, adhere, and remain at the implant surface. Thus, such surface texture-induced microenvironments with stagnated and concentrated blood and proteins may explain the successful, contact osteogenesis or osseointegration. The effects of hydrophilic titanium surfaces on the recruitment of blood, proteins, and cells have been reported extensively [55,56,57,58,59,60,61,62,63,64,65,66,67,68,69,70,71]. The present study has revealed that a similar effect can occur by surface texturing.

Here, we provide a comprehensive visualization and quantification of movement and distribution of blood and protein regulated by nano-topography alone or a combination of nano- and meso-topography, and in doing so, we establish new baseline knowledge during the biological process of osseointegration. While CFD modeling has provided the opportunity to investigate the effects of surface hydrophilicity/hydrophobicity [51], here we extend the utility of this model to another surface property of implants, texture/topography. The CFD model used in this study is new in the fields of implant and bone studies. Therefore, the role of blood and protein flow in initiating and achieving osseointegration has been unexplored or overlooked. The considerable modulation of blood and protein flow by surface topography found in this study will be of great significance in future implant studies. The advantage of CFD models in biological research includes the simulation of liquid, drug, and protein flow. For instance, it is near impossible to examine the blood flow potentially influenced by implant shape and topography in vivo. The CFD model established in this study proved its usefulness and sufficient sensitivity for future studies. The drawback of CFD modeling includes the limited interpretation of data due to the relatively short time frames that can be analyzed. In addition, the model may not represent the actual implant and host environment. For instance, the present model only had three implant threads, which was less than ordinary dental implants. Although blood flow can be diverse or random inside the bone marrow, the present model had blood influx only from the implant apex and adjacent bone. The implant-bone gap designed in this study was 0.3 mm, which may not be standard in regular implant placement. Also, it is difficult to validate the results from CFD models with in vivo events or previous studies. Indeed, as mentioned earlier, this was the first study examining blood flow around differently textured dental implants.

The CFD analysis is rapid and low-cost and can be applied to countless surface topographies as well as the optimization and development of micro- and macroscopic implant designs in the future. For macroscopic morphology, the height and pitch of screw threads can be optimized for better blood and protein recruitment. For micro-scale morphology, studying the effect of different surface roughness will be a priority to optimize the micro-texture. Further, the combined effects of meso-, micro-, and nano-topography will be interesting [15,19,24]. We also believe CFD models are useful to test the host condition. Osseointegration does not solely depend on the implant surface property but also surgical technique and bone quality of the recipient. CFD models with different blood supplies and protein densities and other diverse anatomical conditions can simulate unfavorable host conditions. Thus, CFD models are expected to be used to better understand the biological events around implant surfaces and to improve and optimize future implant design.

## 5. Conclusions

The objective of this study was to examine blood and protein movement around implants with three different surface topographies using a CFD model. The three surface topographies compared were an amorphous surface, a surface with nano-topography alone, and a surface with meso- and nano-topography. Blood and protein flow was analyzed in the implant interface zone (closest to implant surface) and the thread zone (an area within implant screw threads).

1. Implants with nano-topography recruited more blood and protein to the implant interface compared with amorphous implants, and implants with hybrid topography further increased recruitment, with particularly efficient recruitment from the thread zone to the interface zone.
2. The blood movement was significantly slower at the implant interface compared with in the thread zone for all the topographies.
3. Blood movement was slowest at the meso–nano hybrid interfaces and fastest at the amorphous interfaces. The blood velocity at the interface was 3- and 4-fold lower for the hybrid topography compared with the nano-topography and amorphous surfaces, respectively.
4. Fibrinogen recruitment to the implant interface was the most efficient around the meso–nano topography and least efficient around the amorphous surface.

Thus, the present study revealed the impact of surface topography on the recruitment and retention of blood and proteins around implants. In particular, co-texturization with meso- and nano-topography created the most favorable microenvironment.

## Figures and Tables

**Table 1:** Summary of blood and fibrinogen dynamics regulated by surface topography.

	Blood Plasma Density		Blood Velocity		Blood Vector		Fibrinogen Density		Interfacial Recruitment Efficiency	
	Interface	Thread	Interface	Thread	Interface	Thread	Interface	Thread	Blood Plasma	Fibrinogen
Amorphous	+/−	+/−	+/−	+/−	Up and In	Up and In	+/−	+/−	+/−	+/−
Nano-trabeculae	+	++	Slow	Fast	Up and Out	Up and In	+	++	−	+/−
Hybrid (meso-spike and nano-trabeculae)	++	+	Very slow	+/−	Up and In	Up and In	++	+	+	++

+/−: Standard. +: High. ++: Very high. −: Low. Up: Upbound. Down: Downbound. In: Inbound. Out: Outbound.

## Data Availability

Data availability upon request from author.
